# Monomethylarsonous Acid (MMA^III^) Has an Adverse Effect on the Innate Immune Response of Human Bronchial Epithelial Cells to *Pseudomonas aeruginosa*

**DOI:** 10.1371/journal.pone.0142392

**Published:** 2015-11-10

**Authors:** Emily G. Notch, Britton C. Goodale, Roxanna Barnaby, Bonita Coutermarsh, Brent Berwin, Vivien F. Taylor, Brian P. Jackson, Bruce A. Stanton

**Affiliations:** 1 Department of Microbiology and Immunology, Center for Environmental Health Sciences, Geisel School of Medicine at Dartmouth, Hanover, New Hampshire, United States of America; 2 Department of Physical and Biological Sciences, Western New England University, Springfield, Massachusetts, United States of America; 3 Department of Earth Sciences, Dartmouth College, Hanover, New Hampshire, United States of America; University of Alabama at Birmingham, UNITED STATES

## Abstract

Arsenic is the number one contaminant of concern with regard to human health according to the World Health Organization. Epidemiological studies on Asian and South American populations have linked arsenic exposure with an increased incidence of lung disease, including pneumonia, and chronic obstructive pulmonary disease, both of which are associated with bacterial infection. However, little is known about the effects of low dose arsenic exposure, or the contributions of organic arsenic to the innate immune response to bacterial infection. This study examined the effects on *Pseudomonas aeruginosa* (*P*. *aeruginosa)* induced cytokine secretion by human bronchial epithelial cells (HBEC) by inorganic sodium arsenite (iAs^III^) and two major metabolites, monomethylarsonous acid (MMA^III^) and dimethylarsenic acid (DMA^V^), at concentrations relevant to the U.S. population. Neither iAs^III^ nor DMA^V^ altered *P*. *aeruginosa* induced cytokine secretion. By contrast, MMA^III^ increased *P*. *aeruginosa* induced secretion of IL-8, IL-6 and CXCL2. A combination of iAs^III^, MMA^III^ and DMA^V^ (10 pbb total) reduced IL-8 and CXCL1 secretion. These data demonstrate for the first time that exposure to MMA^III^ alone, and a combination of iAs^III^, MMA^III^ and DMA^V^ at levels relevant to the U.S. may have negative effects on the innate immune response of human bronchial epithelial cells to *P*. *aeruginosa*.

## Introduction

According to the World Health Organization (WHO) and the Agency for Toxic Substances and Disease Registry (ATSDR) arsenic is the number one contaminant of concern for human health worldwide [1]. Hundreds of millions of people worldwide are exposed to arsenic via their drinking water, many at doses higher than the WHO maximum contaminant level of 10 ppb [2]. The United States Geological Survey has reported that more than 25 million people in the U.S. are exposed to well water with arsenic concentrations exceeding 10 ppb, the current EPA standard for public water supplies [3,4]. Although the level of arsenic in water in the U.S. is generally lower than in Asia and South America, levels of arsenic in well water in Maine have been measured as high as 3,100 ppb and blood levels of total arsenic ranging from 0.23 to 8.58 ppb have been measured in a rural North Carolina population indicating exposure via food and drinking water [3,5].

Recently, rice, and rice-based products including toddler formulas and energy bars have been identified as major contributors to arsenic exposure [6–9]. For people with low levels of arsenic exposure via drinking water, food constitutes 54–80% of the exposure risk [10]. Many rice and rice based food products contain significant amounts of organic species of arsenic [9,11]. This is cause for concern as little is currently known about the impact of organic forms of arsenic exposure on human health. Some studies have indicated that trivalent DMA and MMA are more toxic than inorganic arsenic, however these studies were done at high concentrations in animal models, and toxicity varies by species and oxidation state [12]. For example, MMA^III^ (180 ppb) has been shown to inhibit cholesterol biosynthesis and inhibit steroid receptor binding to DNA response elements in mammalian cells [13,14]. However, to our knowledge there are no studies that have examined the effects of low levels of organic arsenic exposure on the innate immune response. Although animal studies examining immune response have been conducted using low levels of arsenite in the drinking water, because arsenite is metabolized in the liver it is not possible to determine if the reported effects are the result of arsenite, MMA or DMA [15].

Arsenic exposure in Asia and South America has been linked with a variety of lung diseases including pneumonia, chronic obstructive pulmonary disease (COPD), bronchiectasis, chronic bronchitis and lung cancer [16–18]. Pneumonia, bronchiectasis and COPD are frequently associated with the opportunistic pathogen, *Pseudomonas aeruginosa* (*P*. *aeruginosa*), one of the leading causes of nosocomial infections throughout the world [19,20]. While it has been shown that low-level exposure to arsenic in zebrafish and mice alters the immune response to viral and bacterial pathogens, little is known about the mechanisms by which this alteration occurs [15,21]. In addition, nothing is known about effects of low levels of arsenic on the innate immune function of human lung. Accordingly, the goal of this study was to examine the impact of low levels of arsenic and methylated metabolites on *P*. *aeruginosa* induced cytokine secretion in primary human bronchial epithelial cells (HBEC).

*P*. *aeruginosa* lung infections stimulate the secretion of several cytokines by HBEC including IL-8, IL-6, CXCL1 and CXCL2 [22–24]. These cytokines are chemotactic, and recruit neutrophils and macrophages to the lungs, which are the primary phagocytic cells in the lung responsible for bacterial clearance and killing [20,25]. In addition, these phagocytes produce inflammatory cytokines in response to *P*. *aeruginosa* that elicit many of the key responses that are critical to normal clearance of *P*. *aeruginosa* infection [22,26]. However, during chronic and excessive pulmonary infection and inflammation, the prolonged cellular stimulation and presence of inflammatory cytokines leads to lung damage [19,27]. By contrast, an inappropriately low immune response to *P*. *aeruginosa* reduces the recruitment and activation of neutrophils and macrophages, which reduces the ability to clear *P*. *aeruginosa* from the lungs and causes irreversible pulmonary damage [27]. Thus, an appropriate cytokine response to bacterial infection is required to resolve infections with minimal damage to the lungs.

Since little is known about the effects of low dose arsenic exposure, or the contributions of organic forms of arsenic exposure to the innate immune response to *P*. *aeruginosa* infection, the goal of this study was to examine the effects on *P*. *aeruginosa* induced cytokine secretion by HBEC by inorganic sodium arsenite (iAs^III^) and two major metabolites, monomethylarsonous acid (MMA^III^) and dimethylarsenic acid (DMA^V^), at concentrations relevant to blood levels measured in the U.S. population. Exposure of bronchial epithelial cells to ingested arsenic occurs via the blood *in vivo*. Primary HBEC from several individuals were exposed to arsenic concentrations relevant to blood levels in cell culture media.

## Methods

### Chemicals and Bacterial Strains

DMA^V^ and iAs^III^ were purchased from Sigma (St. Louis, MO). MMA^III^ was synthesized at the Synthetic Chemistry Facility Core at University of Arizona according to previously published methods [28,29]. Fresh concentrated stocks of iAs^III^, DMA^V^ and MMA^III^ (10 ppm) were made in distilled, ultrapure water. Concentrated stocks were diluted to working stock solutions (1 ppm) in cell culture media with fresh dilution stock for each experiment. To minimize degradation of MMA^III^, stocks were maintained at -20°C and fresh dilutions were used for each experiment per standard protocols from the synthetic chemistry facility core at University of Arizona [29]. Purified CXCL2 was purchased from R&D Systems (Minneapolis, MN) and diluted in distilled, ultrapure water. *P*. *aeruginosa* (PAO1) was grown in rich medium (Luria broth, LB, Invitrogen Grand Island, NY) at 37°C. Overnight cultures were washed three times and then added to the HBEC cells at a multiplicity of infection (MOI) of 25 as previously described [30].

### Cells

Primary cultures of human bronchial epithelial cells (HBEC) were purchased from Lonza (Hopkinton, MD). Cells from four individual donors were used for these studies. All donors were Caucasian males between the ages of 32–40 years old. HBEC cells were passaged a maximum of two times. All experiments were repeated with each individual donor a minimum of twice, each with a different passage. Control and *P*. *aeruginosa* exposure were repeated with each cell passages along with arsenic exposure. HBEC were maintained at 37°C with 5% CO_2_ in bronchial epithelial growth media (BEGM) supplemented with bovine pituitary extract, insulin, hydrocortisone, human epithelial growth factor, epinephrine, transferrin, retinoic acid, triiodothryonine and gentamycin from Lonza, according to the manufacturers instructions. Cells were plated in coated 6 well tissue culture plates at 5x10^5^ cells per well. HBEC were exposed to either iAs^III^, MMA^III^ or DMA^V^ in cell culture media at concentrations from 0.5–10 ppb for 6 days, with media renewal every 2 days. Media contained either iAs^III^, MMA^III^ or DMA^V^ as appropriate at each change. Dose ranges were chosen based on blood levels measured in those with drinking water containing 90 ppb in Bangladesh and US blood levels [3,31]. In some experiments HBEC were exposed to a combination of iAs^III^ (1.25 ppb), MMA^III^ (1.25 ppb) and DMA^V^ (2.5 ppb) or iAs^III^, (2.5 ppb), MMA^III^ (2.5 ppb) and DMA^V^ (5 ppb), to mimic blood levels of 5 ppb and 10 ppb total arsenic. These ratios reflect levels measured in blood [31]. After 6 days, cells were exposed to vehicle or *P*. *aeruginosa* at a MOI of 25 for 1 hour. After PAO1 exposure, HBEC were washed to remove *P*. *aeruginosa* using cell culture media containing 75 μg/mL gentamycin to kill any adherent *P*. *aeruginosa* as *P*. *aeruginosa* exposure for longer than 1h caused significant cell death [30]. After washing, HBEC were incubated without *P*. *aeruginosa* for 5 hours to allow cells to elaborate an immune response. Supernatant was collected to measure cytokine release and cells were lysed to isolate total RNA. All experiments were repeated a minimum of two times with each individual donor. In all donors examined the responses were similar in direction (i.e., increase, decrease or no change), but varied in the absolute changes in cytokine secretion.

To determine if the arsenic induced changes in cytokine secretion by HBEC were biologically relevant, THP-1 cells, a monocyte cell line, purchased from ATCC (Manassas, VA), were exposed to purified CXCL2 at concentrations secreted by HBEC or conditioned media from HBEC experiments. After exposure to CXCL2 or HBEC conditioned media, IL-1β production was measured by ELISA. THP-1 cells were grown in RPMI-1640 with 10% FBS and penicillin and streptomycin. Cells were plated at 1x10^6^ cells per well in 6 well plates and differentiated to macrophages with PMA (20 ng/mL for 48h, Sigma St. Louis, MO) [32]. Cells were then exposed to 150, 500 or 1000 pg/mL purified CXCL2 (R&D Systems, Minneapolis, MN) in standard cell culture media for 24h. In conditioned media experiments, THP-1 cells were plated at 5x10^5^ cells per well in 12 well plates and differentiated to macrophages with PMA as described above. THP-1 cells were then exposed to conditioned media from HBEC experiments diluted 1:3 in standard cell culture media for 24h to ensure that THP-1 cells remained healthy and cytokine response levels were in the linear range. Conditioned media from a minimum of 3 different HBEC donors exposed to 10 ppb MMA^III^, 10 ppb iAs^III^ or 10 ppb total arsenic with and without *P*. *aeruginosa* were used in two replicate THP-1 wells per treatment.

### Cytotoxicity

Lactate dehydrogenase (LDH) release by HBEC was used to assess cytotoxicity of all treatments and was measured using the Promega CytoTox 96 Non-Radioactive Cytotoxicity assay per manufacturers instructions (Madison, WI).

### Measurement of intracellular arsenic

To determine if HBEC metabolize arsenic and to measure the intracellular concentration of iAs, MMA or DMA, HBEC were exposed to 10 ppb or 50 ppb of iAs^III^, MMA^III^ or DMA^V^ for either 2 hours (50 pbb) or 7 days (10 ppb), times that are adequate to metabolize arsenic [33]. Thereafter, HBEC were washed on ice with PBS five times. Cells were lysed with 0.1% Triton-X, and spun at 14,000xg for 20 minutes to pellet cells debris. Speciation analysis of arsenic was done by anion exchange chromatography coupled to ICP-MS and detected iAs^III^, iAs^V^, but only oxidized organic species MMA^V^ and DMA^V^ [34].

### Cytokines

IL-8, IL-6, CXCL1, CXCL2 and IL-1β secretion was measured by ELISA (PromoKine Heidelberg, Germany).

### RNA Isolation

Total RNA was isolated from HBEC after exposure to MMA or a combination to all three species with and without exposure to *P*. *aeruginosa* as described above. Total RNA was isolated with the miReasy kit (Qiagen, Valencia, CA) according to manufacturers instructions. Briefly, cells were lysed in phenol and then chloroform was added for phase extraction. The aqueous phase was mixed with ethanol to precipitate RNA. RNA was cleaned up on a glass fiber filter, and washed three times prior to elution. RNA was eluted in nuclease free water and stored at -80°C until time of use. RNA integrity and concentration was assessed using micro-capillary electrophoresis on an Agilent 2100 Bioanalyzer. RNA was compared to a RNA ladder with 6 RNA transcripts of varying sizes and known concentration of 150 ng/mL. RNA quality was verified by observation of corresponding 18 S and 28 S peaks on the electropherogram. Only intact RNA was used for further analysis.

### qPCR

For quantitative PCR (qPCR) cDNA was synthesized from1 μg of total RNA using Retroscript Reverse Transcriptase (Ambion, Austin, TX) with random decamers. TaqMan Gene Expression Assays for human IL8 (TaqMan Gene Expression Assays, Inventoried assay ID Hs00174103), human IL-6 (TaqMan Gene Expression Assays, Inventoried assay ID Hs00985639), human CXCL1 (TaqMan Gene Expression Assays, Inventoried assay ID Hs00605382) and human CXCL2 (TaqMan Gene Expression Assays, Inventoried assay ID Hs00601975) were purchased from Applied Biosystems (ABI, Foster City, CA). Amplicons were sequenced to verify products. Triplicate reactions containing 100ng cDNA from each sample were amplified with an initial denaturing at 95°C for 10 min, followed by 40 cycles of 15 s at 95°C and 1 min at 60°C. Transcript abundance was calculated based on serial dilution of a standard curve. The standard curves showed a correlation coefficient close to 1 (*R* ^2^ > 0.95) and were linear over a 4-log range.

### Statistics

Statistical significance was assessed by one-way ANOVA followed by Tukey’s HSD post hoc test. All statistical analysis was done with Prism v5.0 (Graph Pad Software, San Diego, CA). All experiments were repeated a minimum of two times with different passages of each individual donor. Data are presented as the mean ± SEM.

## Results

### HBEC Minimally Metabolize iAs^III^, MMA^III^ or DMA^V^

In order to use HBEC to examine effects of individual species of arsenic, we first conducted studies to examine whether these cells metabolize arsenic. Measurements of intracellular arsenic by ICP-MS in HBEC exposed to 50 ppb iAs^III^, MMA^III^, or DMA^V^ for two hours revealed that HBEC do not metabolize arsenic in this time frame (Table 1). Intracellular concentrations of arsenic varied slightly by donor, thus data in Table 1 are presented as the range of concentrations measured. Unexposed control cells showed very low levels of iAs^V^, which was present in all treatments except DMA^V^. In cells exposed to iAs^III^, both iAs^III^ and iAs^V^ were detected, as expected since these species readily interconvert in a pH dependent manner both extra- and intracellularly [13,35]. However, in cells exposed to MMA^III^ only MMA^V^ could be detected, and in cells exposed to DMA^V^, only DMA^V^ could be detected (Table 1). These results agree with previous studies demonstrating that undifferentiated human bronchial epithelial cells minimally metabolize arsenic [36]. Additionally, HBEC were exposed to 10 ppb iAs^III^ for seven days to more fully examine metabolism in longer exposure conditions. Unexposed HBEC and cells exposed to 10 ppb iAs^III^ had iAs^III^ and MMA^V^ levels that were below detection limits (n = 7). Unexposed control HBEC had iAs^V^ levels of 0.146 ± 0.08 ppb and DMA^V^ levels of 0.034 ± 0.02 ppb (n = 7). HBEC exposed to iAs^III^ had iAs^V^ levels of 0.173 ± 0.09 ppb and DMA^V^ levels of 0.054 ± 0.06 ppb (n = 7). Measured iAs^V^ and DMA^V^ were not significantly different between iAs^III^ exposed and unexposed control cells. These longer exposures agree with the minimal metabolism of HBEC previously reported, and reveal that our model system is suitable for examining individual arsenic species [36].

**Table 1:** Range of intracellular arsenic concentrations measured in HBEC exposed to 50 ppb iAs^III^, DMA^V^ or MMA^III^.

	Intracellular Concentration (ng/g)			
Treatment	iAs^III^	iAs^V^	MMA^V^	DMA^V^
Control (n = 3)	bdl	0.06–0.1	bdl	bdl
iAs^III^ (n = 3)	bdl—3.7	bdl—0.5	bdl	bdl
MMA^III^ (n = 3)	bdl	bdl- 0.4	bdl—0.4	bdl
DMA^V^ (n = 3)	bdl	bdl	bdl	bdl– 0.2

Control indicates unexposed cells. bdl = below detection limit of 0.05ng/g.

### iAs^III^, MMA^III^ and DMA^V^ are not cytotoxic

**Fig 1:**
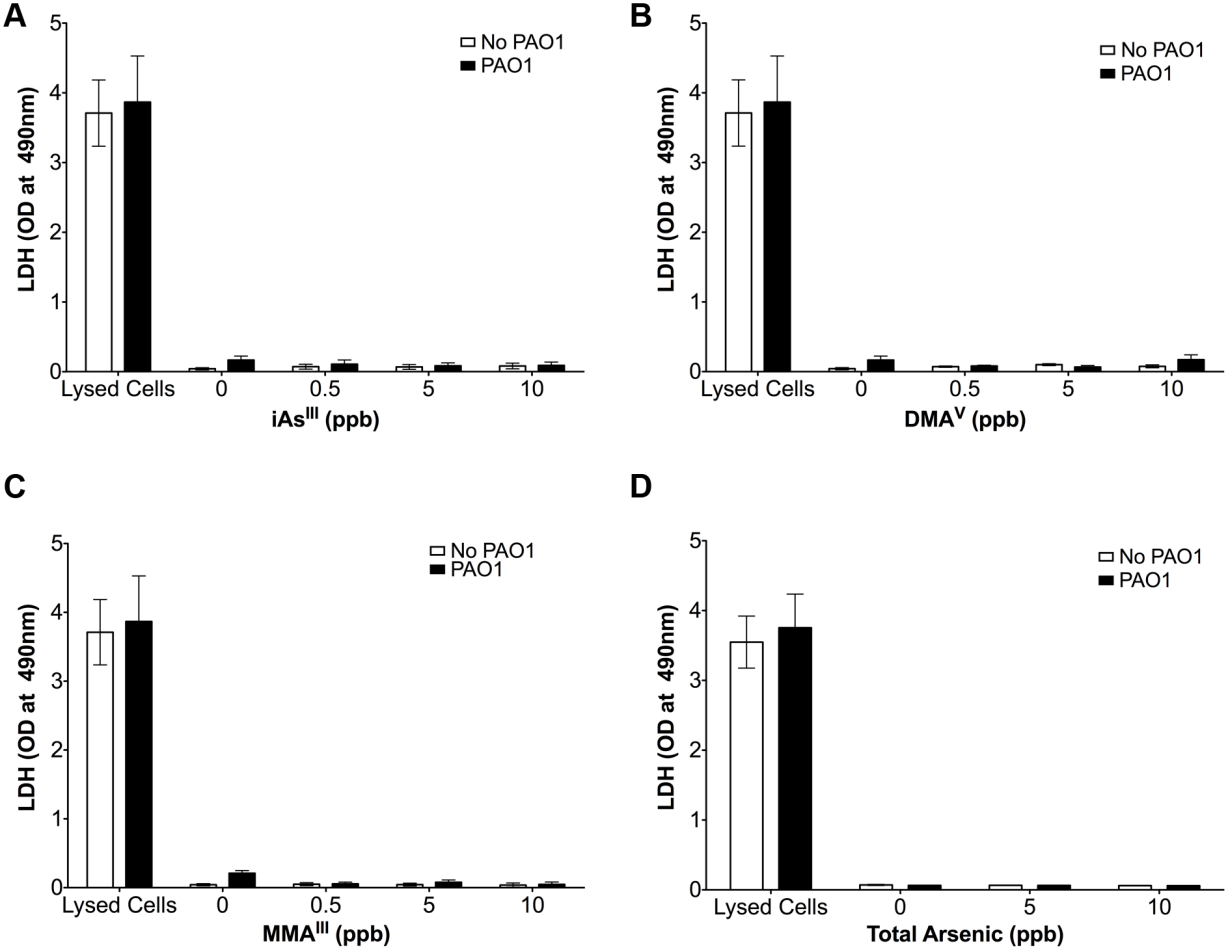
Arsenic exposure does not cause cytotoxicity. LDH release by HBEC was used to assess arsenic cytotoxicity and was measured using the Promega CytoTox 96 Non-Radioactive Cytotoxicity assay per manufacturers instructions. Data reported as optical density (OD, at 490 nm) of the cell culture medium bathing 500K cells. The first two bars represent LDH released by cells lysed by Triton-X (Lysed Cells). Data presented as mean ± SEM. LDH release was not significantly different from 0 in vehicle and arsenic treated cells, with and without *P*. *aeruginosa* exposure. n = 4 donors per treatment group.

Arsenic exposure at high levels can be cytotoxic but less is understood about low doses, in particular when combined with an additional stressor [35,37]. Although HBEC were exposed to very low levels (0.5 to 10 ppb) of iAs^III^, MMA^III^ or DMA^V^, studies measuring LDH were conducted to determine if these arsenic species had cytotoxic effects alone or in combination with *P*. *aeruginosa*. As shown in Fig 1, neither iAs^III^, MMA^III^ nor DMA^V^ alone or in combination with *P*. *aeruginosa* were cytotoxic. *P*. *aeruginosa* alone had no effect on LDH release. This experiment eliminates cytotoxicity as a possible mechanism of action of arsenic and *P*. *aeruginosa* on cytokine secretion by HBEC.

### iAs^III^ and DMA^V^ have no effect on cytokine secretion by HBEC

**Fig 2:**
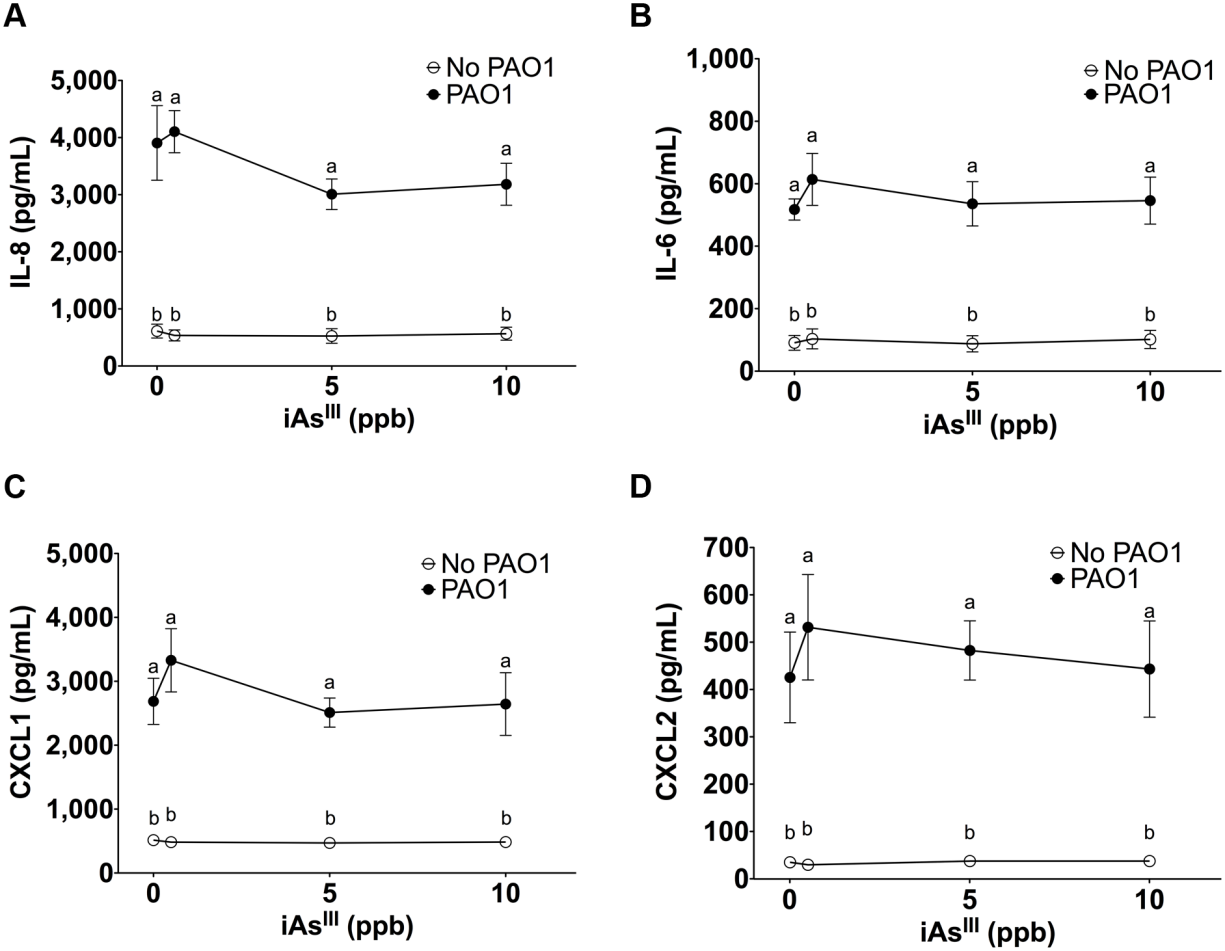
Inorganic arsenic does not alter *P*. *aeruginosa* induced immune response. Cytokine secretion by HBEC exposed to iAs^III^ ± *P*. *aeruginosa*. n = 4 donors for each treatment. Different letters indicate statistically significant treatment means. Data labeled a are not statistically different from each other but are statistically different from data labeled b (p<0.05 as measured by one-way ANOVA). Data with the same letter are not significantly different. (A) IL-8 secretion (B) IL-6 secretion (C) CXCL1 secretion (D) CXCL2 secretion.

**Fig 3:**
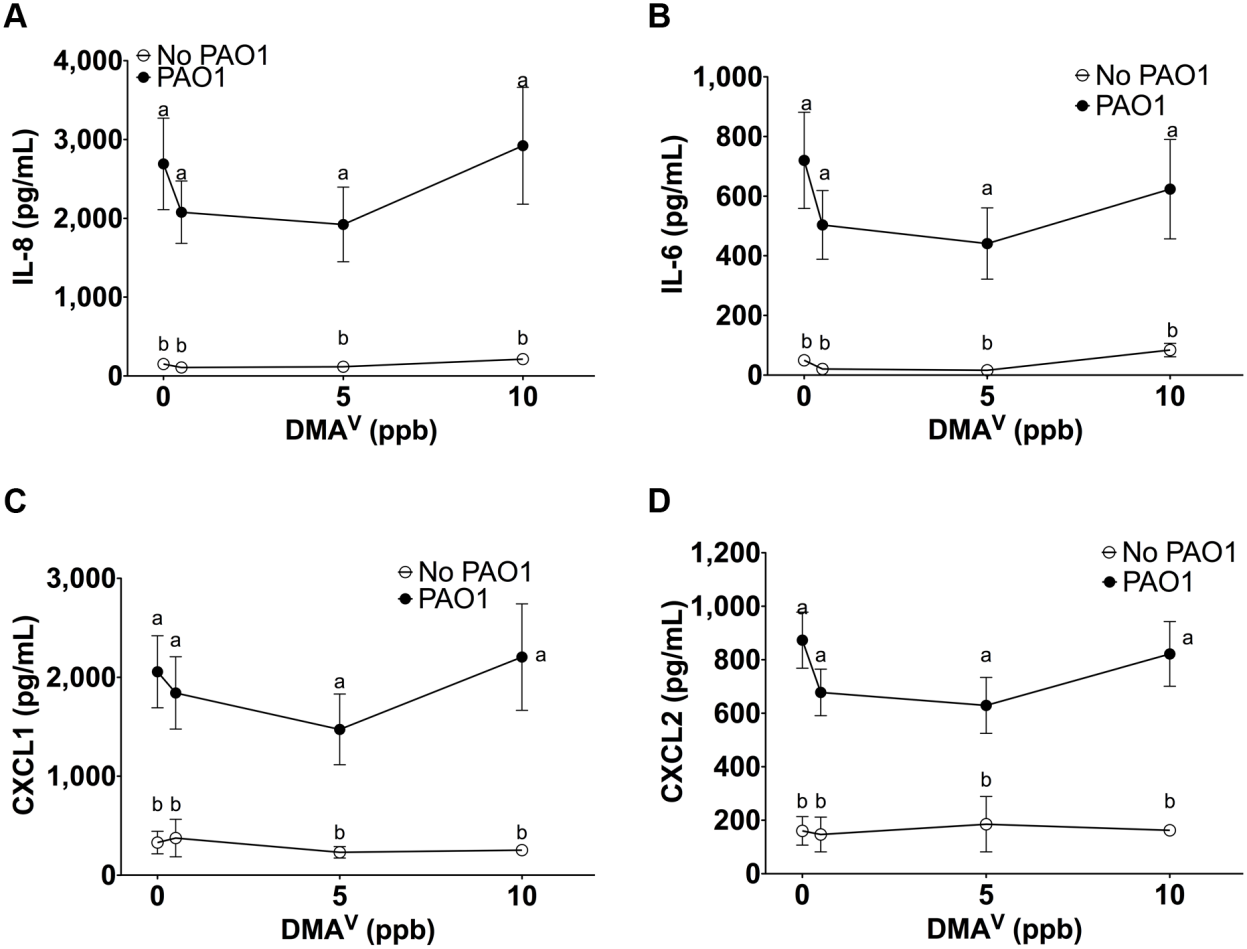
DMA does not alter *P*. *aeruginosa* induced immune response. Cytokine secretion by HBEC exposed to DMA^V^ ± *P*. *aeruginosa*. n = 4 donors for each treatment. Different letters indicate statistically significant treatment means. Data labeled a are not statistically different from each other but are statistically different from data labeled b (p<0.05 as measured by one-way ANOVA). Data with the same letter are not significantly different. (A) IL-8 secretion (B) IL-6 secretion (C) CXCL1 secretion (D) CXCL2 secretion.

iAs^III^ alone (0.5 to 10 ppb) had no effect on IL-6, IL-8, CXCL1 or CXCL2 secretion by HBEC, nor did iAs^III^ alone affect *P*. *aeruginosa* stimulated cytokine secretion (Fig 2). Similarly, DMA^V^ alone (0.5 to 10 ppb) did not significantly affect IL-6, IL-8, CXCL1 or CXCL2 secretion by HBEC (Fig 3).

### MMA^III^ increased *P*. *aeruginosa* stimulated cytokine secretion by HBEC

**Fig 4:**
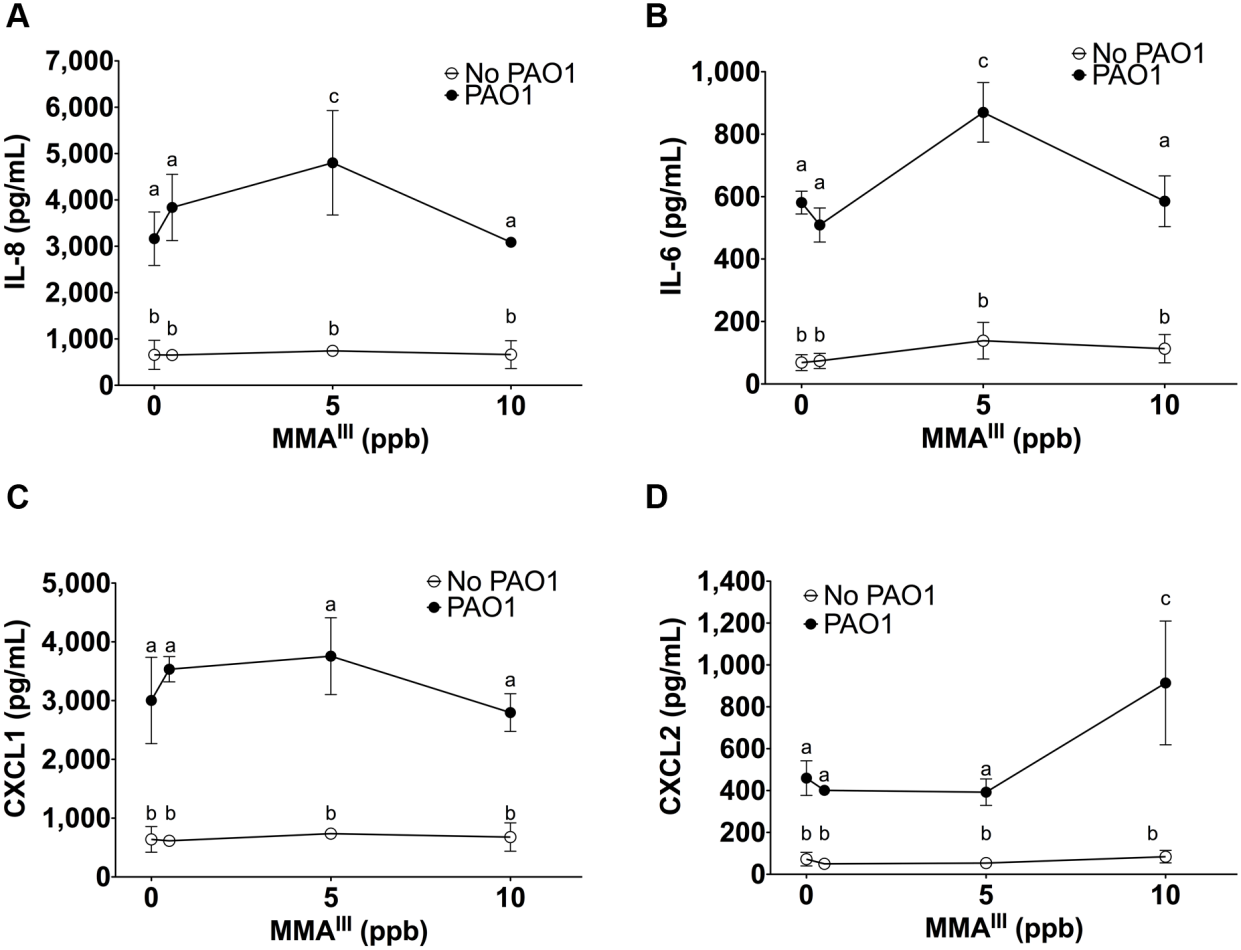
MMA enhances *P*. *aeruginosa* induced immune response. Cytokine secretion by HBEC exposed to MMA^III^ ± *P*. *aeruginosa*. n = 4 donors for each treatment. Different letters indicate statistically significant treatment means. Data labeled a are not statistically different from each other but are statistically different from data labeled b or c (p<0.05 as measured by one-way ANOVA). Data with the same letter are not significantly different. (A) IL-8 secretion (B) IL-6 secretion (C) CXCL1 secretion (D) CXCL2 secretion.

MMA^III^ alone (0.5 to 10 ppb) had no effect on IL-6, IL-8, CXCL1 or CXCL2 secretion (Fig 4). However, 5 ppb MMA^III^ significantly increased *P*. *aeruginosa* stimulated IL-8 and IL-6 secretion in comparison to *P*. *aeruginosa* alone (Fig 4A and 4B). By contrast, 10 ppb MMA^III^ had no effect on *P*. *aeruginosa* stimulated IL-8 and IL-6 secretion (Fig 4A and 4B). MMA^III^ also had no effect on *P*. *aeruginosa* induced CXCL1 secretion at any concentration tested (Fig 4C). Although 0.5 and 5 ppb of MMA^III^ had no effect on *P*. *aeruginosa* stimulated CXCL2 secretion, 10 ppb MMA^III^ increased CXCL2 secretion (Fig 4D). Thus, taken together these data demonstrate that MMA^III^ (5 ppb) enhances *P*. *aeruginosa* induced secretion of IL-8 and IL-6 and that MMA^III^ (10 ppb) enhances *P*. *aeruginosa* induced secretion of CXCL2.

### A combination of iAs^III^, MMA^III^ and DMA^V^ reduced *P*. *aeruginosa* stimulated cytokine secretion

**Fig 5:**
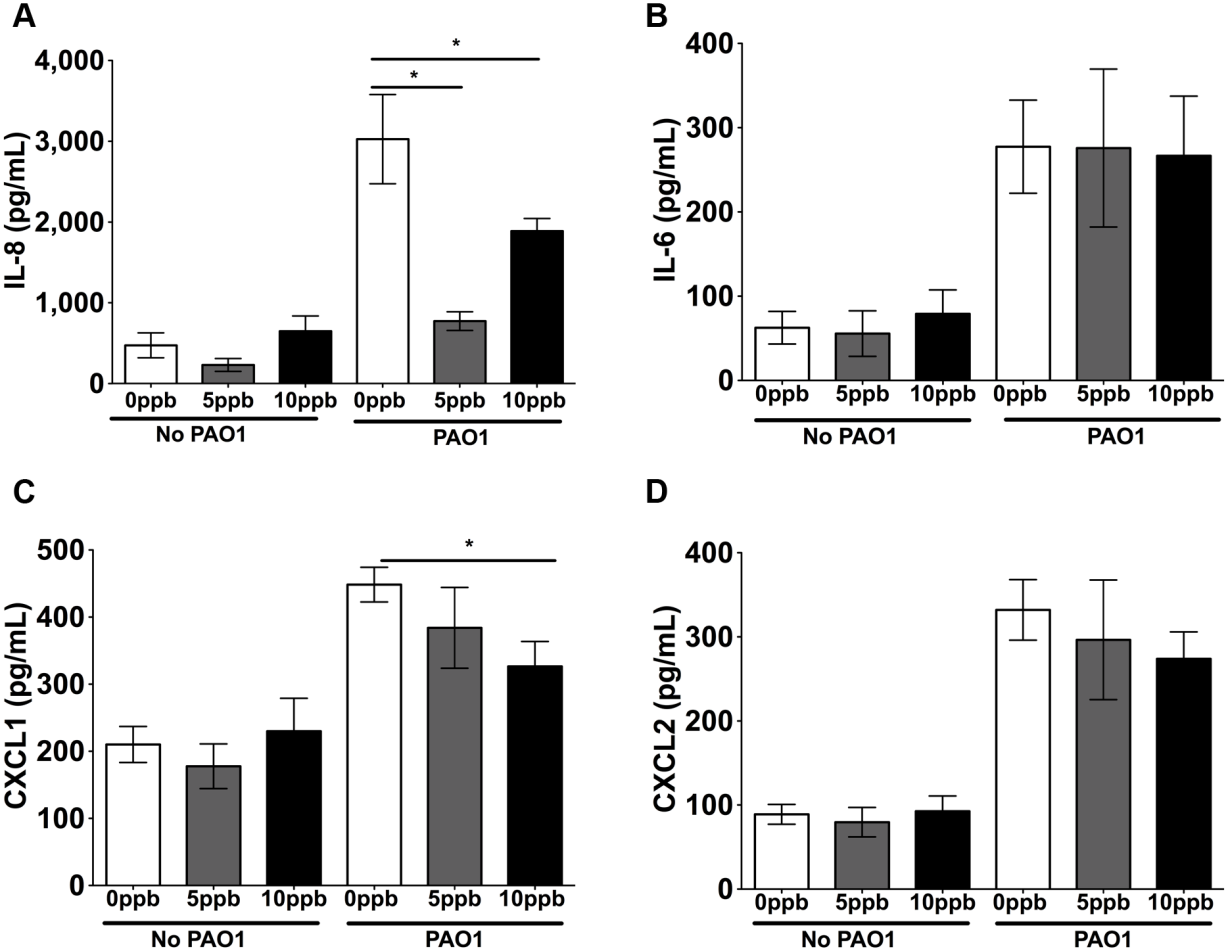
A combination of iAs^III^, MMA^III^ and DMA^V^ reduced *P*. *aeruginosa* stimulated cytokine secretion. Cytokine secretion by HBEC exposed to 5 ppb total arsenic (combination of 1.25 ppb iAs^III^ + 1.25 ppb MMA^III^ + 2.5 ppb DMA^V^) ± *P*. *aeruginosa* or 10 ppb total arsenic (combination of 2.5 ppb iAs^III^ + 2.5 ppb MMA^III^ + 5 ppb DMA^V^) ± *P*. *aeruginosa*. n = 4 donors for each treatment. *p = <0.05 for the indicated comparisons. (A) IL-8 secretion (B) IL-6 secretion (C) CXCL1 secretion (D) CXCL2 secretion.

Since blood of individuals who drink water contaminated with iAs^III^ and iAs^V^ typically contains mixtures of iAs, MMA and DMA, because inorganic arsenic is metabolized in the liver, we conducted studies to examine the effect of a combination of organic and inorganic arsenic at levels measured in blood samples obtained in the U.S. [3,31]. HBEC were exposed to 5 ppb or 10 ppb total arsenic, composed of 50% DMA^V^, 25% MMA^III^ and 25% iAs^III^. Neither 5 ppb nor 10 ppb total arsenic alone had a significant effect on basal cytokine secretion compared to control (Fig 5). Both 5 ppb and 10 ppb total arsenic significantly reduced *P*. *aeruginosa* stimulated IL-8 cytokine secretion (Fig 5A). 5 ppb total arsenic had no effect on *P*. *aeruginosa* stimulated IL-6, CXCL1 or CXL2 secretion (Fig 5B, 5C and 5D), but 10 ppb total arsenic significantly reduced *P*. *aeruginosa* stimulated CXCL1 secretion (Fig 5C).

### IL-1β secretion by THP-1 cells is regulated by cytokines released by HBEC

**Fig 6:**
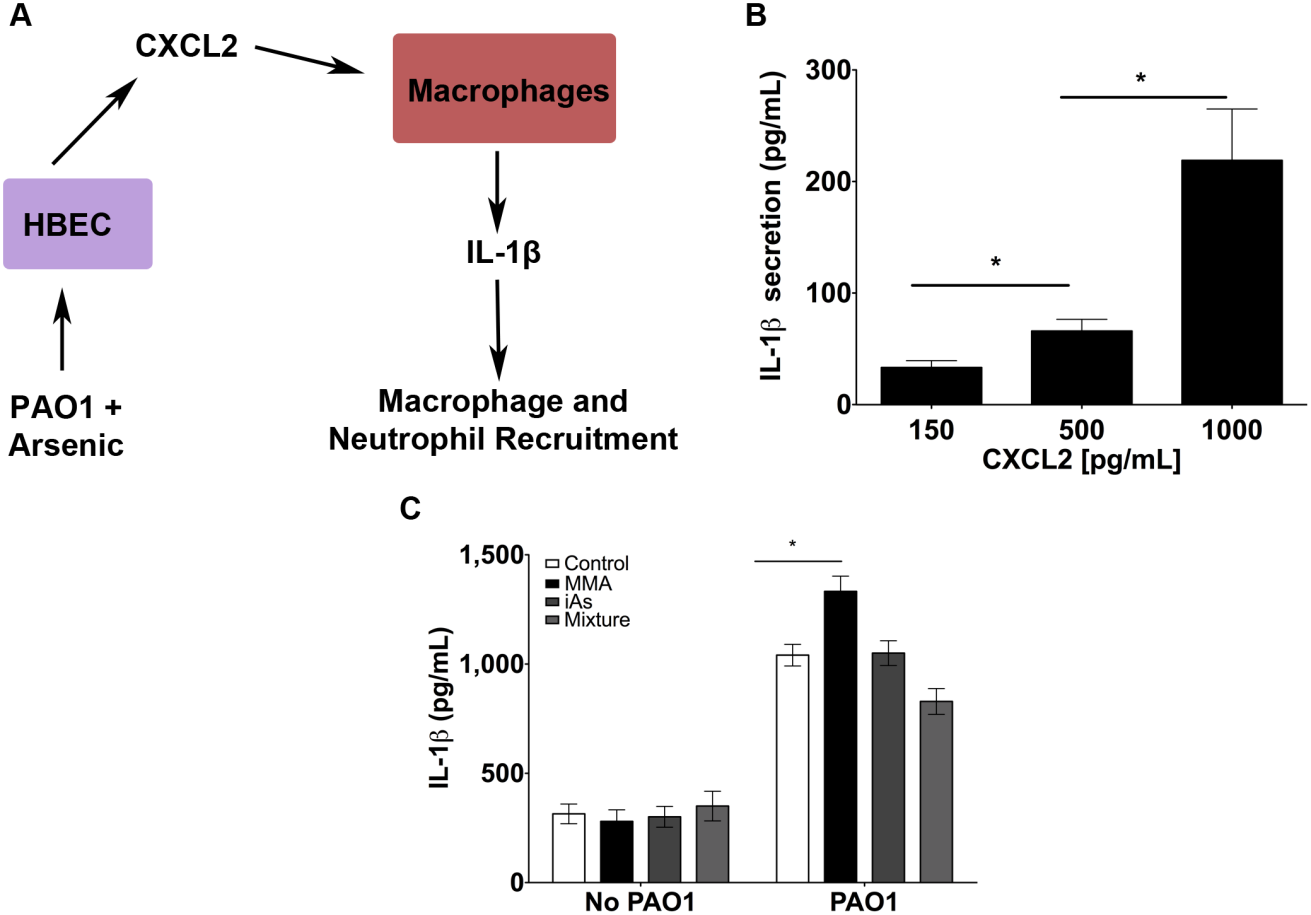
Changes in HBEC cytokine production impacted macrophage IL-1β production. (A) Model of how arsenic exposure alters HBEC production of cytokines, which impacts cytokine secretion by macrophages. (B). IL-1β secretion by THP-1 cells stimulated with purified CXCL2 using the range of concentrations released by HBEC in the present experiments. n = 4 donors. (C) IL-1β secretion by THP-1 cells stimulated with conditioned media from HBEC treated with vehicle (control), 10 ppb MMA, 10 ppb iAs or 10 ppb total arsenic ± *P*. *aeruginosa*. n = 3 donors. *p = <0.05 for the indicated comparisons.

Studies were conducted to determine if the MMA^III^ induced changes in CXCL2 secretion by HBEC had a significant effect on IL-1β production by differentiated THP-1 cells, a model macrophage cell line (Fig 6A). IL-1β secretion by macrophages recruits additional macrophages and neutrophils into the lungs, and is an essential component of the innate immune response to bacterial infection [20]. Thus, studies were conducted to determine if the MMA^III^ induced increase in CXCL2 secretion by *P*. *aeruginosa* exposed HBEC had a significant effect on IL-1β secretion by THP-1 cells. An increase in CXCL2 concentration from 500 to 1000 pg/mL, the concentrations produced by HBEC exposed to *P*. *aeruginosa* and *P*. *aeruginosa* plus 10 ppb MMA^III^, respectively, significantly increased IL-1β secretion (Fig 6B). THP-1 IL-1β release was modest when stimulated with CXCL2 in comparison to stimulation with conditioned media from HBEC.

To further examine the impact of altered HBEC cytokine secretion on macrophage IL-1β secretion, THP-1 cells were exposed to conditioned media from HBEC exposed to 10 ppb MMA^III^, 10 ppb iAs^III^ or 10 ppb total arsenic with and without *P*. *aeruginosa*. Conditioned media from HBEC exposed to 10 ppb MMA^III^, 10 ppb iAs^III^ or 10 ppb total arsenic in the absence of *P*. *aeruginosa* did not significantly alter THP-1 IL-1β production compared to conditioned media from control HBEC (Fig 6C). Conditioned media from HBEC exposed to *P*. *aeruginosa* significantly increased IL-1β secretion by THP-1 cells compared to control conditioned media. Conditioned media from HBEC exposed to *P*. *aeruginosa* plus 10 ppb MMA^III^ significantly increased THP-1 production of IL-1β compared *P*. *aeruginosa* alone. It is important to note that in the studies presented in Fig 6C 10 ppb MMA^III^ increased cytokine (IL-6 and IL-8) secretion by HBEC cells. 10 ppb iAs^III^ did not alter cytokine secretion by HBEC in the presence of *P*. *aeruginosa*. While 10 pbb total arsenic reduced IL-8 and CXCL1 secretions by HBEC, these reductions were not sufficient to significantly alter IL-1β in macrophages. Taken together, these data suggest that the alteration of IL-6 and IL-8 secretion in *P*. *aeruginosa* stimulated HBEC by 10 ppb MMA^III^ will significantly alter IL-1β production by macrophages.

### Neither MMA^III^ nor a combination of iAs^III^, MMA^III^ and DMA^V^ altered cytokine mRNA

**Fig 7:**
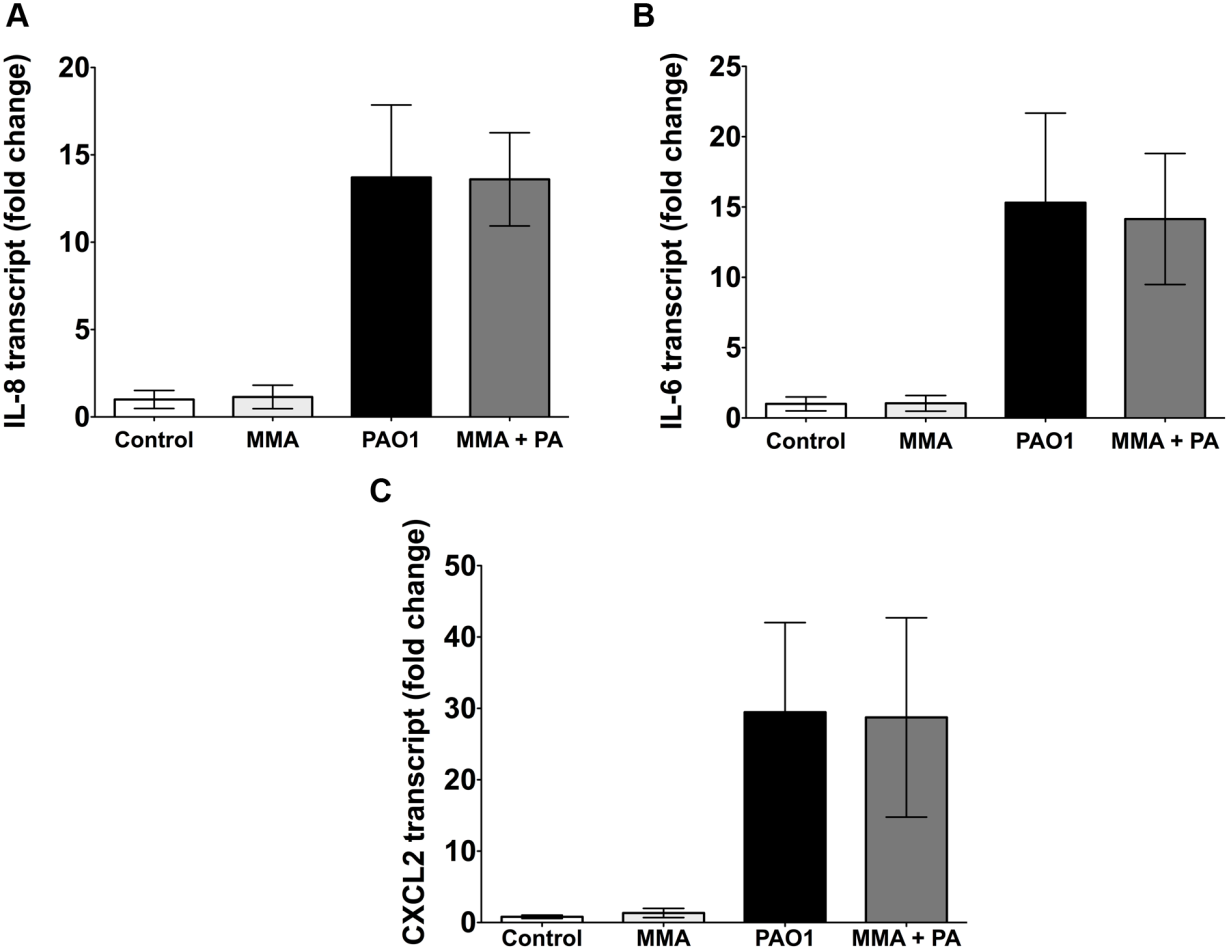
MMA^III^ had no effect on cytokine mRNA levels. MMA^III^ (5 ppb) had no effect on (A) IL-8 or on (B) IL-6 mRNA levels. In addition, MMA^III^ (10 ppb) had no effect on (C) CXCL2 mRNA levels. Thus, the observed increases in IL-8, IL-6 and CXCL2 cytokine levels induced by MMA^III^ were not related to increased mRNA levels. n = 4 donors.

**Fig 8:**
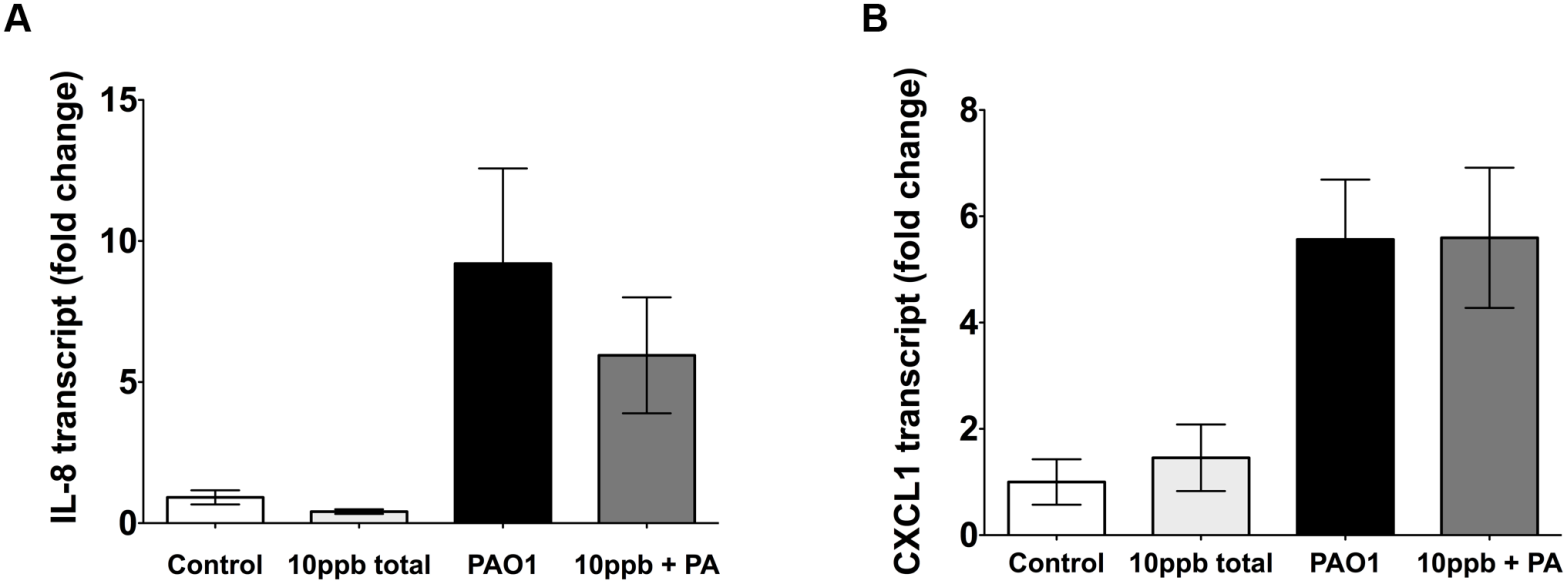
Exposure to mixtures of arsenic species does not alter cytokine mRNA. A mixture of arsenic (10 ppb: 2.5 ppb As^III^, 2.5 ppb MMA^III^ and 5 ppb DMA^V^) had no effect on (A) IL-8 or (B) CXCL1 mRNA. Thus, the observed reductions in IL-8 and CXCL1 cytokine levels induced by the mixture of arsenic species were not related to decreased mRNA levels. n = 4.

To examine whether changes in HBEC cytokine secretion were the result of transcriptional regulation or post-translational modification, cytokine mRNA levels were measured. As shown in Figs 7 and 5 ppb MMA^III^ alone had no effect on IL-8 or on IL-6 mRNA levels (Fig 7A and 7B). In addition, 10 ppb MMA^III^ alone had no effect on CXCL2 mRNA levels (Fig 7C). *P*. *aeruginosa* significantly increased IL-8, IL-6 and CXCL2 transcript levels, but the presence of MMA^III^ did not alter *P*. *aeruginosa* induced mRNA. Thus, the observed increases in IL-8, IL-6 and CXCL2 cytokine levels induced by MMA^III^ were not related to an increase in cytokine mRNA. In addition, studies were conducted to determine if the inhibitory effect of a combination of iAs^III^, MMA^III^ and DMA^V^ (10 ppb) on IL-8 and CXCL1 secretion was mediated by a decrease in mRNA. However, as shown in Fig 8, the combination of iAs^III^, MMA^III^ and DMA^V^ alone had no effect on IL-8 or on CXCL1 mRNA levels (Fig 8A and 8B). *P*. *aeruginosa* stimulated IL-8 and CXCL1 mRNA, but 10 ppb total arsenic did not alter the *P*. *aeruginosa* induced mRNA. Thus, the observed decreased in IL-8 and CXCL1 cytokine levels induced by a combination of iAs^III^, MMA^III^ and DMA^V^ (10 ppb) were not related to decreased mRNA levels of these cytokines.

## Discussion

Arsenic exposure is a global health concern with a variety of deleterious health effects. The immunotoxicity of arsenic is poorly understood and represents an important area of study [17]. Alteration of inflammatory processes, in particular in TNFα and NFκB signaling, has been observed in infants exposed to arsenic *in utero* [38–40]. However nothing is known about the relative contributions of inorganic versus organic species of arsenic to immunotoxicity. To our knowledge this is the first study to examine the impacts of MMA^III^ and DMA^V^, at concentrations that are relevant to the US population, on the innate immune response of HBEC to a bacterial pathogen. The major novel finding is that a combination of 10 ppb total iAs^III^, MMA^III^ and DMA^V^, reflecting blood levels relevant to drinking water exposures, suppressed IL-8 and CXCL1 secretion by HBEC. In addition, MMA^III^ alone exacerbated the immune response of HBEC to *P*. *aeruginosa*. Taken together, these data demonstrate that low levels of arsenic disrupt cytokine secretion by *P*. *aeruginosa* stimulated HBEC.

Results from this HBEC study are similar to research using a co-culture model of Caco-2 cells (a human colon epithelial cell line) and peripheral blood monocyte cells (PBMC) that showed 9 ppb MMA^III^ enhanced LPS induced IL-6 and TNFα release [41]. The same study using Caco-2/PBMC in co-culture also showed that 105 ppb DMA^III^ plus LPS reduced IL-8 and IL-6 release into the apical media in comparison to LPS alone [41]. Interestingly, in contrast to our findings, Caco-2 cells and the Caco-2/PBMC co-culture showed significant release of pro-inflammatory cytokines with exposure to iAs^III^, MMA^III^ or DMA^III^ alone [41,42]. By contrast, HBEC in this study showed low basal levels of pro-inflammatory cytokines regardless of arsenic exposure. These differences are potentially due to higher concentrations of iAs, MMA and DMA used in the Caco-2 study, or may simply represent tissue differences in response to arsenic species [42].

Pro-inflammatory cytokine secretion by bronchial epithelial cells is the first response to bacterial infection [23]. The initial increase in cytokine secretion by HBEC recruits professional immune cells, including macrophages and neutrophils, into the lungs, which secrete copious amounts of cytokines that mobilize additional immune cells to eliminate the bacterial infection [20]. Here we show that MMA^III^ enhances the innate immune response of HBEC, which may increase the production of cytokines by macrophages, and potentially lead to excessive inflammation, which has been shown to produce lung damage [20]. We found that the change in cytokine secretion by HBEC induced by MMA^III^ in this study had significant effects on IL-1β secretion by differentiated THP-1 cells, a model macrophage cell line. While *in vivo* prolonged production of cytokines can result in lung damage, initially this increased cytokine secretion may be a beneficial augmentation, resulting in enhanced recruitment of macrophages and more rapid clearance of pathogens [20]. Further study is required to determine which of these responses are seen with low level MMA^III^ exposure.

Interestingly, 10 ppb total arsenic, a combination of iAs^III^, MMA^III^ and DMA^V^ reflecting relative blood levels after exposure via drinking water, reduced cytokine secretion by HBEC. Reduced cytokine secretion by HBEC would be expected to reduce macrophage recruitment and pathogen clearance. However, the reduced cytokines produced by HBEC after exposure to 10 ppb total arsenic did not significantly reduce IL-1β in THP-1 cells, suggesting that the reduction in IL-8 and CXCL1 will not alter macrophage response. The differences between MMA^III^ alone and the combinations of arsenic species will require further study to understand the specific mechanisms for each type of exposure.

Low dose inorganic arsenic has previously been shown to reduce clearance of pathogens in zebrafish and mouse lung, however similar results have not previously been reported in human cells [15,21]. The published animal studies used a variety of viral and bacterial pathogens including Influenza A (H1N1), snakehead rhabdovirus, and *Edwardsiella tarda* indicating that altered immune response with arsenic can happen with a variety of pathogens [15,21]. Studies related to arsenic in the human lung have shown that arsenic exposure compromises respiratory immune response through several mechanisms including decreased airway epithelial chloride secretion, altered activation of pulmonary alveolar macrophages and impaired wound response resulting in airway remodeling [43–45]. Thus, arsenic has many effects on the innate immune response to bacterial infection.

IL-8 and CXCL2 are transcriptionally regulated by NFκB, and other transcription factors [46,47]. Previous studies have indicated that one mechanism for immunotoxicity of arsenic is through interaction with NFκB; including enhanced NFκB activation by MMA^III^ in uroepithelial cells, and activation of NFκB signaling in cord blood of newborns with *in utero* arsenic exposure [29,39]. However, we did not observe an effect of MMA^III^ or the combination of iAs^III^, MMA^III^ and DMA^V^ on mRNA levels of IL-6, IL-8, CXCL1 or CXCL2, thus, the arsenic induced changes in cytokine production observed in the present study are likely to occur by post-transcriptional mechanisms.

One possible post-transcriptional mechanism is that MMA^III^ may enhance secretion of these cytokines through a change in membrane fluidity. Studies have indicated that low levels of MMA^III^ perturb cholesterol biosynthesis [14]. Additional studies are necessary to examine the distinct molecular mechanisms whereby MMA^III^ and the combination of iAs^III^, MMA^III^ and DMA^V^ at levels found in blood alter *P*. *aeruginosa* induced cytokine secretion in HBEC.

Data from this study show dysregulation of proinflammatory cytokines in HBEC after organic arsenic exposure and that these altered cytokines have downstream effects, altering IL-1β secretion in THP-1 cells. Our data provide insight into the possible mechanisms whereby arsenic exposure increases the relative risk of respiratory infection and COPD, which is associated with chronic bacterial infections, and other non-malignant lung infections [16,48]. Moreover, this study provides important data demonstrating that organic forms of arsenic, at low doses, have negative effects on the innate immune response of human bronchial epithelial cells to *P*. *aeruginosa* infection *in vitro*.
